# MochiView: versatile software for genome browsing and DNA motif analysis

**DOI:** 10.1186/1741-7007-8-49

**Published:** 2010-04-21

**Authors:** Oliver R Homann, Alexander D Johnson

**Affiliations:** 1 Department of Microbiology and Immunology, University of California San Francisco, San Francisco, California, USA; 2 Department of Biochemistry and Biophysics, University of California San Francisco, San Francisco, California, USA

## Abstract

**Background:**

As high-throughput technologies rapidly generate genome-scale data, it becomes increasingly important to visually integrate these data so that specific hypotheses can be formulated and tested.

**Results:**

We present MochiView, a platform-independent Java software that integrates browsing of genomic sequences, features, and data with DNA motif visualization and analysis in a visually-appealing and user-friendly application.

**Conclusions:**

While highly versatile, the software is particularly useful for organizing, exploring, and analyzing large genomic data sets, such as those from deep RNA sequencing, chromatin immunoprecipitation experiments (ChIP-Seq and ChIP-Chip), and transcriptional profiling. MochiView provides an extensive suite of utilities to identify and to explore connections between these data sets and short sequence motifs present in DNA or RNA.

## Background

We describe a versatile tool for visualizing and exploring large genomic data sets, particularly those generated by ***ch***romatin ***i***mmuno***p***recipitation (ChIP). This technique is often used to identify regions of a genome that are bound by a specific transcription factor under a given set of conditions. For those transcription factors that recognize DNA directly, it is often possible, from ChIP data alone, to deduce the range of DNA sequences (the motif) that a given transcription factor recognizes. ChIP relies on cross-linking transcription factors to DNA in living cells, shearing and isolating the DNA, and recovering the DNA cross-linked to a specific transcription factor. The recovered DNA is then analyzed using either tiling microarrays (ChIP-Chip) or sequencing (ChIP-Seq). Both approaches generate a nearly continuous profile of binding enrichment across the genome, with high-density tiling for ChIP-Chip currently being feasible only for smaller genomes, such as those from bacteria or fungi. Several existing genome browsers aid in the visualization and analysis of such data, but few contain tools to easily integrate motif data into the analysis. MochiView (***Mo***tif and ***ChI***P ***View***er) is designed to bridge this gap, providing a highly flexible and intuitive interface that allows one to easily import, visualize, explore, and analyze large sets of data, such as those generated from ChIP experiments.

## Implementation

MochiView is written in Java, and can be used with any operating system that supports Java version 1.6 or higher. To facilitate smooth genome browsing (by caching data) and the import of large files, MochiView requires hardware with a minimum of 1 GB memory. Many genome browsers introduce an extra layer of complexity by requiring the user to install an external database or to store data on a remotely hosted server. MochiView circumvents this problem by transparently incorporating the Java DB database within the software (specific features of the MochiView software design are described in the MochiView manual). The database architecture is designed to scale well even with very large quantities of data; database size is primarily constrained by available hard drive space. In practice, database sizes can range from a few megabytes to many gigabytes in size, depending on genome size and the quantity of data. MochiView can maintain multiple databases, and contains a database import/export utility to facilitate sharing of compressed databases (and plot configurations) between users. Any database can be populated by the user with one or more genomes by importing the genome sequence as one or more FASTA-format files. Additional genome coordinate-based data can then be uploaded in the commonly used GFF, BED, or WIG formats or using MochiView's own custom file formats. Tips for setting up a database are provided on the MochiView website.

## Results and discussion

MochiView serves as both a motif analysis platform and a feature-rich genome browser, and integrates these features to allow the visualization of motifs across a genome plot and the refinement of motif analyses using data imported by the user into the MochiView database (for example, genome alignments, ChIP data, or expression data). While many of the tools provided in MochiView were designed with ChIP-Seq and ChIP-Chip data visualization in mind, the open and flexible data format allows the import and visualization of any data that have a genomic context (for example, high-throughput RNA sequencing data). MochiView is user-friendly, and is accessible to scientists with no programming knowledge. MochiView's many features are extensively documented with a tutorial walkthrough, a detailed manual, and extensive popup text support within the software. While many of MochiView's individual features are available in existing software, no existing software package, to our knowledge, integrates such a large assortment of motif and data analysis utilities together with a highly configurable genome browser in a single desktop application. The most similar existing package, CisGenome [1], provides a greater emphasis on processing of raw ChIP-Chip and ChIP-Seq data and peak-finding, but is limited with respect to the scope and ease of use of the motif and data visualization and analysis options.

### Visualizing data across the genome

**Figure 1:**
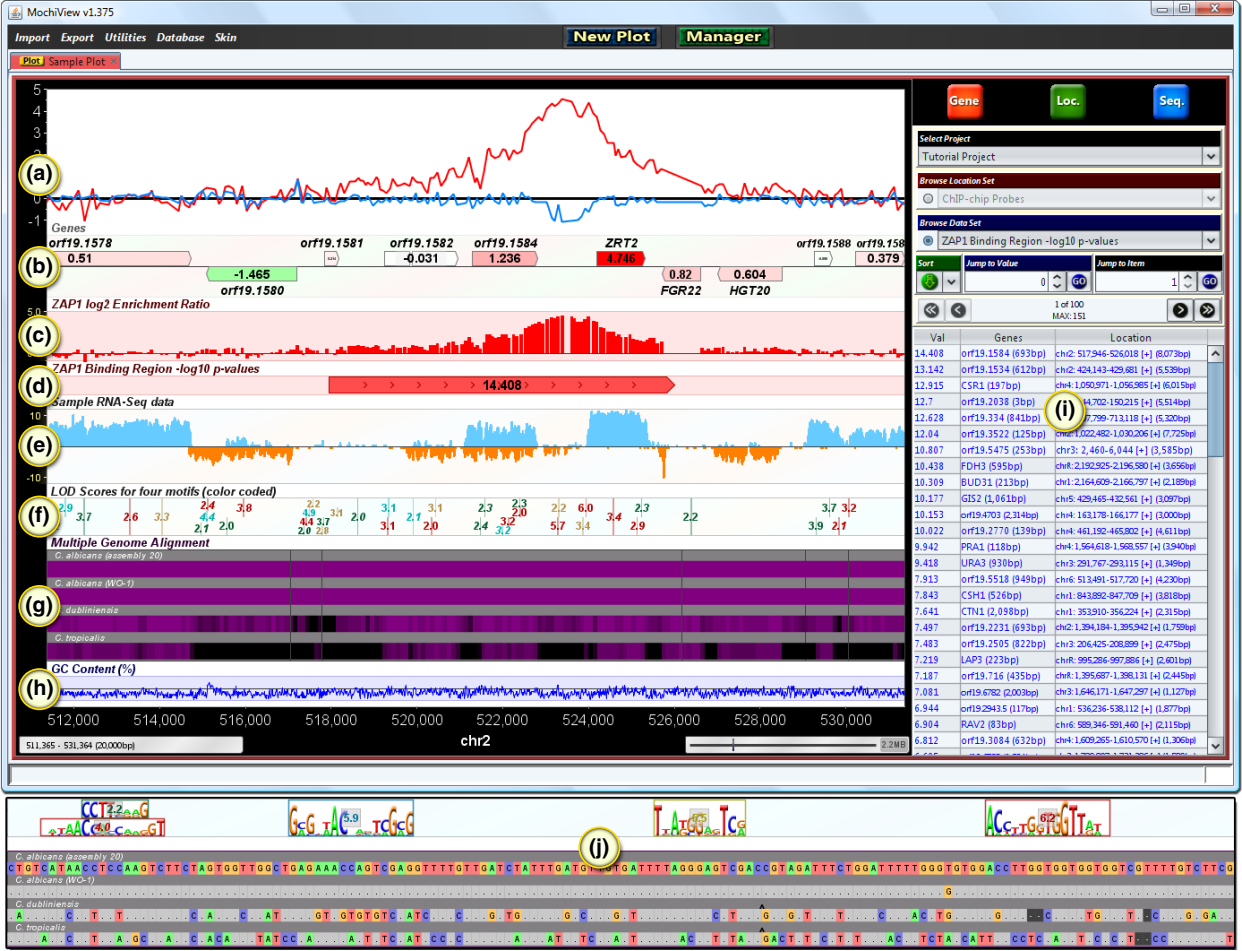
**A sample MochiView screenshot, demonstrating many of the available display formats**. A 20 kb span of the *Candida albicans *genome is displayed. **(a) **Two line graphs utilizing the same y-axis and representing experimental (red) and control (blue) ChIP-chip enrichment data for the Zap1 transcription factor [33]. **(b) **A gene track, including color-coded data representing log_2_-transformed expression values from a microarray experiment. This experiment compares a wild-type strain to a Zap1 deletion strain. Note that red indicates the highest expression change; *ZRT2 *is likely to be a direct target of Zap1. Gene tracks can also display genes containing multiple isoforms and coding- and non-coding exons (not shown). **(c) **A bar graph track, demonstrating an alternate means of displaying the experimental ChIP-chip data represented by the red line graph in (A). **(d) **A region marker track, depicting a ChIP binding region and -log_10_-transformed *P*-value. **(e) **An RNA sequencing track, depicting mock data mapped to the plus strand (blue) and minus strand (orange). **(f) **A motif track, depicting the motif match scores of instances of four different DNA motifs, each assigned to a different color. **(g) **A multiple genome alignment track (several species of yeast), shaded to represent the level of conservation. **(h) **A line graph track, representing the GC-content of the DNA. **(i) **The data browser, which displays the contents of the database in an interactive table. Clicking on a row in the table centers the plot on the corresponding region. **(j) **Additional features become evident as the plot is zoomed in. Shown here are close-ups of the motif and alignment tracks (F and G, respectively).

MochiView uses an integrated local database to manage all of the data imported by the user, such as genome sequences and alignments, gene locations, microarray probe locations, expression data, ChIP data, and motif libraries. As shown in Figure 1, MochiView allows many types of data to be displayed along the genome (the x-axis of the plot) in easily customized plots. Open plot tabs persist when the software is closed and reopened, and the display settings can be saved for later use. While the core design of MochiView's plots was inspired by the UCSC Genome Browser project [2], MochiView places an added emphasis on aesthetics, data browsing, and plot interactivity, and provides a rich interface for configuring plot layout.

Landmarks across a genome (such as the locations of microarray probes) are displayed by *region markers *(Figure 1D). Overlapping markers can be displayed as *stack tracks *with one region marker positioned above the other. Numerical data, such as ChIP-Chip enrichment levels, can be displayed in MochiView using line or bar plots. These data sets can be plotted on a common y-axis (Figure 1A) or each set can be plotted on its own y-axis (Figure 1C, E, H). Alternatively, numerical data can be displayed as text on a region marker (Figure 1D), and the marker can be colored according to the value (a useful means, for example, of visualizing expression data on genes; see Figure 1B). Sequences matching DNA motifs are identified using a user-defined scoring threshold and are displayed in additional tracks (Figure 1F). Multiple genome alignments, either genomes from closely related species or from individuals of the same species, can also be displayed (Figure 1G), providing the means to quickly visualize whether a motif match is conserved across closely related genomes (phylogenetic footprinting; see Cliften *et al. *[3] and Kellis *et al. *[4]), or whether it varies in interesting ways.

### Tools for browsing and interacting with data in a plot

MochiView provides tools for browsing the genome by sequence or by data set. The sequence browser can be used to search and highlight specific DNA sequences, degenerate DNA sequences (using symbols established by the International Union of Pure and Applied Chemistry), and direct or inverted repeats, with or without gaps. The data browser (Figure 1I) allows the user to sort and search any data set and rapidly jump from location to location across the genome using hotkeys. For example, this feature allows the user to quickly browse among regions of ChIP enrichment above a user-specified threshold value to rapidly visualize the most significant binding regions. These can then be searched for matches to a particular DNA motif.

MochiView plots are interactive and allow smooth panning along chromosomes and smooth zooming in and out. As one continues to zoom in, the DNA sequence itself eventually becomes visible. Virtually every element in a plot provides descriptive popup text, and annotation can be added to locations within tracks. In addition, clicking on any item in a plot copies the sequence to the clipboard, a useful tool for quickly capturing sequences for use in another application. To aid the user in filtering large sets of data, an *Edit Mode *track can be created and used to toggle a region marker between three states (true/false/undecided). For example, this feature is useful for flagging and ignoring likely false positives in a set of ChIP binding data.

MochiView's motif and multiple genome alignment tracks (Figures 1F and 1G, respectively) are also interactive. Motif tracks show either the match scores of motif instances (distant zoom) or the motif logo itself (close zoom; top of Figure 1J). Double-clicking the motif instance opens a window juxtaposing the motif logo with the actual genome sequence. Multiple genome alignments are displayed as either an overview shaded by conservation level (distant zoom) or as the specific aligned sequences, including inserts and gaps (close zoom; bottom of Figure 1J). Clicking on the alignments, or on the carets representing inserts in the alignment, copies the regional alignment to the clipboard.

### ChIP analysis highlights many of MochiView's utilities

MochiView can serve as a central hub for data storage and visualization, from which data can easily be imported and exported for manipulation with other applications. In addition, MochiView contains a number of specific tools designed to analyze genomic and motif data. While a description of all of the utilities provided in MochiView is beyond the scope of this article, we discuss a few of them in the context of analyzing ChIP data for proteins that recognize specific DNA sequences. We focus on two stages of analysis: (1) visualization of the primary ChIP data and assessment/refinement of the *binding region calls*, and (2) identification and characterization of regulatory motifs found within the refined binding regions. We define a *binding region *as a set of genomic coordinates that identify the boundaries of a region of ChIP DNA enrichment, typically associated with some measure of confidence, such as a *P*-value. Obviously, proper control experiments are crucial to evaluate the biological relevance of a binding region, a topic discussed in more detail below.

### Visualizing and refining ChIP data in MochiView

The first step of ChIP data analysis in MochiView is typically the import of raw data (ChIP-Chip enrichment or ChIP-Seq reads) as well as the binding region calls (*peak calls*). MochiView does not supply a comprehensive binding region assignment algorithm (a more limited peak extraction/refinement utility is provided), as approaches to calling binding regions are constantly being refined; moreover, the approaches for calling peaks vary with the platform used to analyze the precipitated DNA. For example, Agilent supplies peak-calling software optimized for its array design. It is, however, straightforward to import peak-calling results from existing software using MochiView's import utilities, which support several different file formats. For small genomes, it is also possible to hand-curate ChIP data in MochiView, bypassing the peak-calling programs entirely.

Once the relevant raw data (ChIP-Chip enrichment or ChIP-Seq reads) and binding region calls are imported, MochiView can be used to visualize them in the context of other genomic information. For example, ChIP data can be viewed in a plot in conjunction with control ChIP experiments, gene expression data, sequence GC-enrichment, histone modifications, and motifs. The *snapshot *utility allows the user to create individual images (or a single pdf) of the plot centered at every binding region in the data set. This feature is particularly useful for records in laboratory notebooks or figures for manuscripts.

For those data sets with a manageable number of binding regions, it is possible to visually inspect each binding region and eliminate clear false positives (and re-evaluate possible false negatives) that result from the limitations of binding site detection algorithms. Since MochiView can display multiple data sets on the same y-axis, the user can easily overlay multiple replicates of experimental ChIP data as well as control data sets (for example, ChIP in a deletion or RNAi-depleted strain or in a strain lacking the epitope tag targeted for immunoprecipitation). These data can then be quickly surveyed using the data browser and an *Edit Mode *track, and binding regions considered spurious (for example, those also observed in control experiments) or unreliable (for example, those observed in only one experimental replicate) can be flagged and then filtered using one of MochiView's data refinement utilities.

MochiView provides numerous additional utilities for the analysis and manipulation of sets of locations. Set operation utilities can take the union, intersection, or subtraction of two location sets, thus providing a simple mechanism for manipulating positional data. For example, the user can merge the binding region calls of experimental replicates, take the intersection of binding regions with promoter regions, take the intersection of sets of ChIP experiments performed with different transcription factors, or easily eliminate binding region calls that overlap with regions found in a control experiment. Another utility assigns binding regions to one or more genes (based on user-defined criteria), and another surveys whether these genes are enriched for Gene Ontology (GO) terms (using an approach based on the software GO TermFinder [5]). Thus, within minutes of importing ChIP data into MochiView, a user can obtain an overview of the cellular processes and genes predicted to be regulated by the transcription factor of interest. An important goal of many ChIP-Chip and ChIP-Seq experiments is the identification of the DNA motif recognized by the transcription factor of interest, and, as described next, MochiView provides numerous tools for the discovery, validation, and comparison of motifs.

### Identifying and analyzing motifs in MochiView

**Figure 2:**
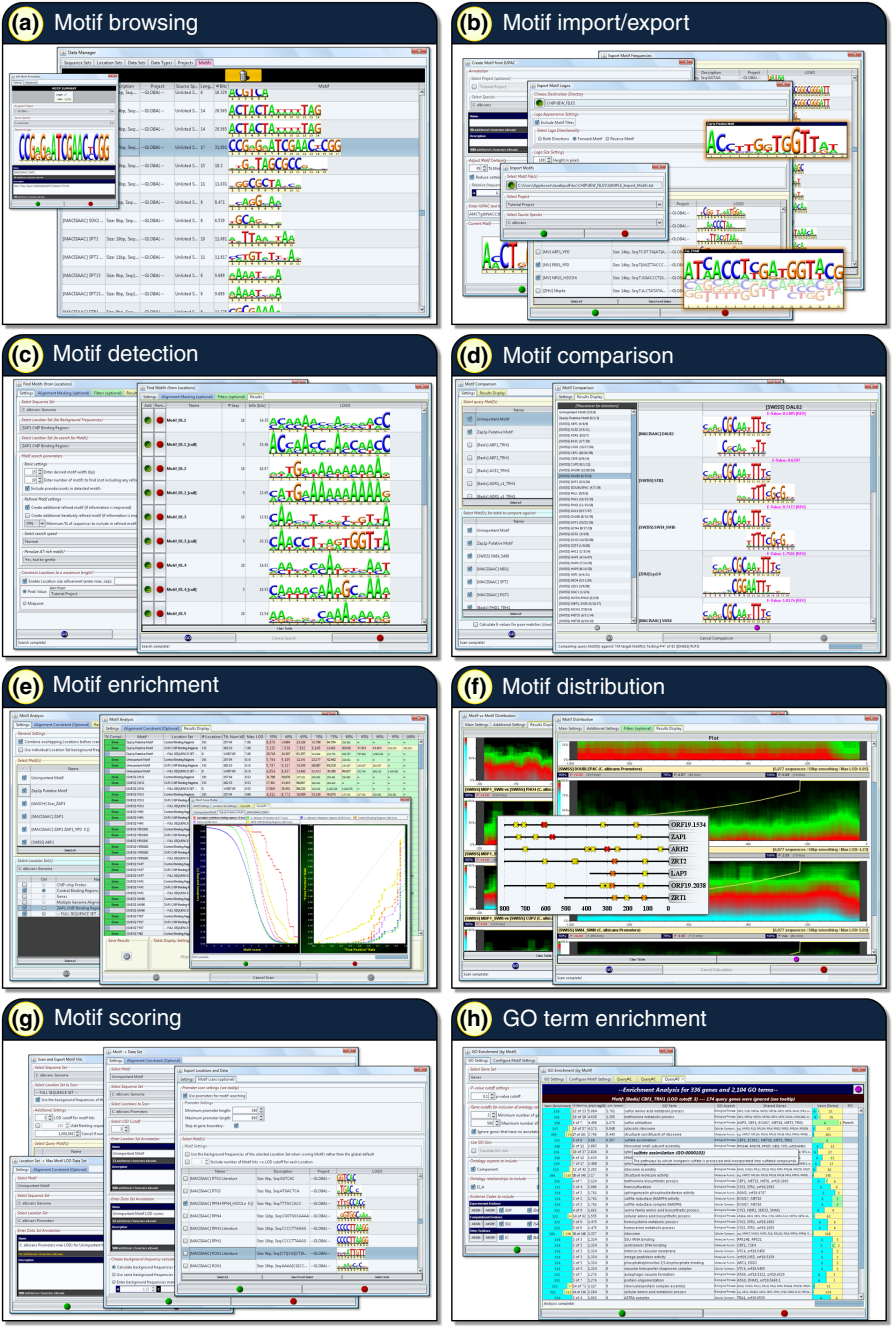
**An overview of MochiView's regulatory motif analysis and management tools**. (a) MochiView provides a simple interface for browsing and annotating a motif library. **(b) **MochiView provides numerous utilities for importing and exporting motif frequency matrices and logos, including support for motifs based on degenerate DNA sequences, frequency matrices, or affinity matrices (as produced by the program MatrixREDUCE [7]). **(c) **MochiView contains a motif detection utility that can identify *de novo *motifs enriched in user-defined regions. **(d) **A motif comparison tool identifies similarities between newly discovered motifs and those in the motif library. **(e) **Two utilities are provided for analyzing motif enrichment in sets of user-defined regions. **(f) **Utilities are provided for detecting non-random distribution of motifs relative to either a set of user-defined locations (for example, start codons or peaks of ChIP enrichment) or strong instances of another motif (for example, co-occurring motifs that are typically separated by a 25 bp gap). **(g) **Several utilities are provided for scoring motifs against user-defined regions. For example, it is relatively simple to output a file containing the top motif score upstream of each gene for every motif in the library. **(h) **Enrichment for Gene Ontology terms can be determined for genes with upstream sequence that contains a strong instance of a motif.

We use the term *motif *to mean a set of short DNA sequences represented by a position-specific weight matrix, and define a *motif match *as a particular DNA sequence in a genome that is statistically similar to a motif. Several options are provided for scoring a DNA sequence for matches to a motif, including logarithm of odds (LOD) scores (reviewed in [6]), affinity scores (for affinity motifs generated by MatrixREDUCE [7]), and *P*-values derived from LOD scores (using the compound importance sampling algorithm of Barash et al. [8]). In addition to finding particular matches to a motif within a sequence, MochiView can also generate a cumulative motif enrichment score for a full sequence using either a simple cumulative LOD score or a Hidden Markov Model approach (w-score, as described by Sinha et al. [9]). Figure 2 provides an overview of the many utilities provided in MochiView for the visualization, management, and analysis of motifs. (These tools are not specifically tied to ChIP-Chip and ChIP-Seq analysis; they can be used in any context.) Motifs in MochiView are visualized as logos, using a format based on the sequence logo design originally described by Schneider and Stephens [10]. The MochiView database provides a convenient means to maintain and annotate a library of motifs (Figure 2A), and these motifs can easily be exported as frequency matrices or logos (Figure 2B). Several motif libraries, derived from a broad range of organisms including yeast [11-19], nematode [20], human [18,19,21,22], and mouse [18,19,23-26], are provided at the MochiView website in a format this is simple to import into MochiView. This collection includes one of the largest curated motif libraries, over 1,300 motifs, provided courtesy of the JASPAR database [18,19]. Additional motifs devised by the user are also easy to import into MochiView.

MochiView provides a motif detection utility (Figure 2C) that can identify motifs *de novo *using a Gibbs sampling technique (based on algorithms described by Thijs *et al. *[27] and the BioJava [28] online cookbook; implementation details are provided in the manual). The user can limit a search to specific locations (for example, binding region calls from a ChIP experiment) or search the upstream regions of a list of specific genes. It is also possible to specify that a motif occurrence must be conserved across closely related genomes. The features of MochiView also allow the user to rapidly conduct motif searches based on more complex queries. For example, the user could chain together utilities to search for motifs in the portions of binding regions that (1) overlap with intergenic regions, (2) are within 200 bp of a peak of ChIP enrichment, (3) do not overlap with areas of enrichment in the control experiment, and (4) neighbor a gene that changes expression when the transcription factor of interest is deleted (or reduced in expression by RNAi) or overexpressed. As an alternative to the built-in motif detection utility, the user can also export a set of sequences of interest (for example, those that lie within 200 bp of a peak of ChIP enrichment), apply a different motif-finding algorithm, and import the results back into MochiView. MochiView supports multiple motif file formats, including the output of the commonly used motif detection applications MEME [29] and Bioprospector [30].

Often, the first step in the analysis of a newly discovered motif is a determination of whether the motif resembles any known motifs. Motif libraries, such as those provided at the MochiView website, can be compared against newly discovered motifs using the motif comparison utility (Figure 2D), which generates a similarity metric based on the algorithm used by the software TomTom [31]. This utility allows rapid determination of whether a discovered motif is novel, previously identified, or closely related to a motif of a different species.

Another common query in motif analysis is the extent to which a motif is enriched in the DNA precipitated in a given ChIP experiment (or set of experiments). In other words, how well can the motif predict the ChIP data? The motif enrichment utilities (Figure 2E) allow rapid assessment of motif enrichment at incremental score cutoffs for sets of locations such as binding regions or intergenic regions. To assess their significance, the levels of enrichment can be compared to those of a set of control locations (for example, comparison of upstream regions that include ChIP peaks versus those that do not). This analysis can also be conducted on every motif in the library, allowing the user to identify all known motifs that are enriched in the locations of interest.

Motif analysis often identifies several candidate DNA motifs that may be recognized by the transcription factor of interest. In the simplest cases, where the transcription factor directly recognizes a motif, the motif is predicted to lie under the center of the peak of ChIP enrichment. In other cases, a motif may be significantly enriched in a set of binding regions, not because it is recognized by the transcription factor of interest, but rather because it is bound by a different protein that regulates a similar set of genes. These alternatives can be tested using MochiView's motif distribution utilities (Figure 2F), which test for non-random positional distribution using a statistical test for non-uniform distribution described by Casimiro *et al. *[32]. These utilities can also identify non-random spacing between genomic matches to DNA motifs (for example, two DNA motifs, either the same or different, with matches that are typically separated by a 30 to 50 bp gap).

Once a compelling motif has been identified from a set of ChIP data, the motif can be explored using the MochiView motif scoring utilities (Figure 2G) and the plot browser to identify instances of a motif that occur in intergenic regions but not within the binding regions called by the ChIP-analysis algorithm. Such analysis can reveal whether the motif is necessary and sufficient to describe the binding of the transcription factor of interest. For example, such analysis may identify a set of genes that is likely to be controlled by the transcription factor but is not bound by the protein under the conditions or in the cell types used for the ChIP analysis.

We described above how MochiView's GO term enrichment utility could connect ChIP data to specific cellular processes. This same strategy can be used to search the upstream regions of genes for strong matches to a motif and associate that motif with one or more GO terms (Figure 2H). This approach can provide insight into the biological role of the transcription factor and further validate the motif's biological relevance.

## Conclusions

In summary, MochiView was developed to solve problems we encountered in our basic research efforts, allowing us to integrate different types of genomic data and analyses in a single format where biological correlations and insights *popped *out from the screen. We believe the software will be useful to members of many other basic research laboratories who have encountered similar challenges when interpreting and analyzing data on a genomic scale.

## Availability and requirements

**Project name**: MochiView.

**Project home page**: http://johnsonlab.ucsf.edu.

**Operating system(s)**: Platform independent.

**Programming language**: Java.

**Other requirements**: Java 1.6 or higher, minimum 1GB memory, 1024 × 768 or higher screen resolution.

**License**: MochiView is available in source and executable forms, without fee, for academic, non-profit and commercial users.

**Any restrictions to use by non-academics**: None beyond the general restriction against redistribution in the license.

## Abbreviations

BED: ***B***rowser ***E***xtensible ***D***ata; ChIP: ***ch***romatin ***i***mmuno***p***recipitation; ChIP-Seq: ChIP analyzed using DNA ***seq***uencing; ChIP-Chip: ChIP analyzed using tiling microarrays; GFF: ***G***eneral ***F***eature ***F***ormat; GO: Gene Ontology; WIG: ***W***iggle format.

## Authors' contributions

ORH designed and wrote the software, with support from ADJ. Both authors contributed to the writing of the manuscript. All authors read and approved the final manuscript.
